# Effects of Cu and Ag Elements on Corrosion Resistance of Dual-Phase Fe-Based Medium-Entropy Alloys

**DOI:** 10.3390/ma16083243

**Published:** 2023-04-20

**Authors:** Jianjun Liu, Yanchun Zhao, Ruonan Hu, Minya Zhang, Yutian Ding

**Affiliations:** 1 State Key Laboratory of Advanced Processing and Recycling of Non-Ferrous Metals, Lanzhou University of Technology, Lanzhou 730050, China; 2 Wenzhou Pump and Valve Engineering Research Institute, Lanzhou University of Technology, Wenzhou 325105, China

**Keywords:** medium-entropy alloy, dual-phase, microstructure, corrosion resistance

## Abstract

The effect of adding elements to promote phase separation on the functional properties of medium-entropy alloys has rarely been reported. In this paper, medium-entropy alloys with dual FCC phases were prepared by adding Cu and Ag elements, which exhibited a positive mixing enthalpy with Fe. Dual-phase Fe-based medium-entropy alloys were fabricated via water-cooled copper crucible magnetic levitation melting and copper mold suction casting. The effects of Cu and Ag elements microalloying on the microstructure and corrosion resistance of a medium-entropy alloy were studied, and an optimal composition was defined. The results show that Cu and Ag elements were enriched between the dendrites and precipitated an FCC2 phase on the FCC1 matrix. During electrochemical corrosion under PBS solutions, Cu and Ag elements formed an oxide layer on the alloy’s surface, which prevented the matrix atoms from diffusing. With an increase in Cu and Ag content, the corrosion potential and the arc radius of capacitive resistance increased, while the corrosion current density decreased, indicating that corrosion resistance improved. The corrosion current density of (Fe_63.3_Mn_14_Si_9.1_Cr_9.8_C_3.8_)_94_Cu_3_Ag_3_ in PBS solution was as high as 1.357 × 10^−8^ A·cm^−2^.

## 1. Introduction

As a result of structural fluctuations and differences at the interfaces, such as grain boundaries and phase boundaries, traditional alloys exhibit relatively high Gibbs-free energy. Therefore, corrosion usually occurs preferentially at the grain boundaries and phase boundaries [1]. Medium-entropy alloys (MEAs) with a multi-component solid solution structure have only 2–4 main elements, with a configuration entropy range of 1R–1.5R [2]. A solid solution structure facilitates the even distribution of alloying elements during the formation process. The dispersed distribution of atoms has a pinning effect, which ensures that the material has good strength, hardness, and plastic toughness. A medium-entropy alloy’s multi-component composition has a cocktail effect and increases lattice distortion.

Medium-entropy alloys are developed based on high-entropy alloys (HEAs), a new class of alloys containing two to four major elements with a mixed entropy of 1R–1.5R. The entropy and cocktail effects of medium/high-entropy alloys yield a stable structure, allowing the optimization of corrosion resistance through element regulation [3,4,5,6]. Among them, Fe- and Ti-based MEAs have the most promising application prospects due to their excellent mechanical properties, and their corrosion behavior has attracted much attention. A single-phase medium-entropy alloy has a stable solid solution structure, which avoids the formation of micro-galvanic cells between different phases and has good corrosion resistance. However, the corrosion behavior of a dual-FCC phase medium entropy alloy, which was demonstrated to improve the mechanical behavior and other functional properties such as the antibacterial paramagnetic properties, is rarely studied [7,8,9]. When Ti, Cr, Ni, Co, Mn, Cu, Ag, and other elements exist in the entropy alloy, they first form a dense oxide film on the surface, which effectively prevents corrosion while increasing the alloy’s potential [10,11,12]. Liu et al. [13] prepared the Co_1−x_CrFeNi_1+x_Cu_y_ high-entropy alloy by vacuum arc melting. The microstructure exhibited phase separation behavior, i.e., the Cu-rich phase and the Cu-poor phase. In a 3.5 wt% NaCl solution, a Cu_2_O film with poor corrosion resistance is formed on the surface of the Cu-rich phase, which reduces the resistance and induces pitting corrosion. Due to a certain potential difference between the two regions, the Cu-rich phase is preferentially corroded. Meanwhile, increasing the Ni content and decreasing the Co content are beneficial to improving the solid solubility of Cu in the alloy, reducing the segregation of Cu at the grain boundary, and improving the corrosion resistance of HEAs. Li et al. [14] studied the effect of changes in Cu content on the corrosion resistance of the high-entropy alloy Cu_x_Cr_2_Fe_2_Ni_3_Mn_2_Nb_0.4_Mo_0.2_ (x = 0,0.1,0.2). The results show that with the increase in Cu content, the microstructure of the alloy gradually changes from a mixed structure of FCC and BCC to a single FCC structure. Additionally, the corrosion rate of the alloy first decreases and then increases. A small amount of Cu can improve the passivation ability of the alloy, thereby improving its corrosion resistance. Hsu et al. [15] studied the corrosion behavior of an FeCoNiCrCu_x_ high-entropy alloy in a 3.5% NaCl solution. They found that the local potential difference caused by the segregation of the alloy’s Cu element caused corrosion of the primary battery, and along with the increased Cu content, the alloy’s corrosion resistance decreased. Furthermore, both Ag and Cu are located in the IB group of the periodic table of elements, and the heat mixing enthopy $\Delta H_{Fe-Ag}^{mix}\;$of which is also positive, suggests that Ag may have similar effects as the Cu element on corrosion properties, which are rarely reported.

For this study, the medium-entropy alloy Fe_63.3_Mn_14_Si_9.1_Cr_9.8_C_3.8_ was used as the matrix, and the thermodynamic parameters of the alloy’s system were calculated by adding different amounts of Cu and Ag elements. The effects of alloy composition on the microstructure and phase formation were studied. An electrochemical workstation was used to study the corrosion behaviors of the medium-entropy alloys in phosphate-buffered saline solution (PBS solution), and the alloys’ corrosion resistance was analyzed by combining electrochemical corrosion curves, corrosion morphology, and XPS detection to investigate corresponding corrosion mechanisms.

## 2. Materials and Methods

In this study, the nominal components of ingots, Fe_63.3_Mn_14_Si_9.1_Cr_9.8_C_3.8_, (Fe_63.3_Mn_14_Si_9.1_Cr_9.8_C_3.8_)_(100−x)_Cu_x_(x = 2,4), and (Fe_63.3_Mn_14_Si_9.1_Cr_9.8_C_3.8_)_(100−2x)_Cu_x_Ag_x_(x = 2,3), medium-entropy alloys, were prepared by mixing the constituent elements with purities > 99.99 (weight percent) in a high-frequency induction vacuum furnace using a water-cooled Cu crucible under an argon atmosphere. Each ingot was re-melted at least six times to ensure homogeneous microstructures, and then sucked up by negative pressure into the copper mold for casting and forming. The alloys’ phases and a cross-section close to the surface were studied using an X-ray diffractometer (XRD, D/max-2400) with Cu-Kα radiation (40 kV and 30 mA). Phase identification was performed using JADE 9.5.1 from Materials Data Inc. and the 2012 PDF4+ database from the International Center for Diffraction Data (ICDD) database. Additionally, using FeCl_3_-HCl as the etchant, the microstructures for microscopy were revealed by thermal field-emission scanning electron microscopy (SEM, JSM-5600LV).

The μAutolab type III electrochemical workstation was used to study electrochemical corrosion behaviors (the working electrode was the alloy sample, the auxiliary electrode was a Pt sheet, and the reference electrode was Ag|AgCl|Cl^−^). The corrosion medium was a PBS solution. Electrochemical impedance spectroscopy (EIS) was performed, and dynamic potential polarization curves were plotted for the tested alloys. Samples’ open circuit potentials (φcorr−t curve) were measured before electrochemical testing; when the change in a sample’s φcorr value was less than 1 mV within 100 s, then the sample’s φcorr could be considered stable. The EIS sweep frequency ranged from 10 mHz to 1 kHz, and the perturbation potential was 10 mV. The scanning range of the potentiodynamic polarization curve was −1.0VSCE–1.0VSCE, and the scanning speed was set to 0.5 mV/s. All tests were repeated at least three times. The XPS measurements were performed in an Escalab 210 spectrometer (VG Instruments) using non-monochromatized Al Kα radiation from a twin Mg/Al anode operating at 300 W. The hemispherical energy analyzer was operated in CAE mode with a constant pass energy of 20 eV.

## 3. Results

### 3.1. Thermodynamic Parameters of Fe-Based Medium Entropy Alloys

The thermodynamic parameters of the base alloy and the alloys with different proportions of Cu and Ag elements were calculated.

According to Boltzmann’s equations [16,17], it is assumed that there are N atoms mixed in an alloy, where *n_0_*, *n_i_*, …, *n_r_* are the numbers of different elements and *k* is the Boltzmann constant; then, the mixing entropy Δ*S*_mix_ of an alloy is:

(1) $$\Delta S_{mix}=kln(\frac{N!}{n0!n1!\ldots nr!}).$$

When the atomic ratios are non-equal:

(2) $$\Delta S_{mix}=-R\sum_{i=1}^{i}x_{i}lnx_{i},\;$$

where *x_i_* is the atomic percentage of components and *R* is the gas constant. The mixing enthalpy Δ*H_mix_* can be calculated by using the mixture enthalpy $\Delta H_{AB}^{mix}\;$ of a binary system [18]:

(3) $$\Delta H_{mix}=\sum_{i=1,i\neq j}^{N}4\Delta H_{AB}^{mix}x_{i}x_{j}.\;$$

Moreover, the parameter *Ω*, which can combine the effects of Δ*S_mix_* and Δ*H_mix_* on the stability of a multicomponent solid solution, is defined as [19,20,21]:

(4) $$\Omega =T_{m}\Delta S_{mix}/\left|\Delta H_{mix}\right|,$$

(5) $$T_{m}=\sum_{i=1}^{n}x_{i}T_{i},$$

(6) $$\Delta H_{mix}=\sum_{i=1,i\neq j}^{n}\Omega_{ij}c_{i}c_{j},$$

where *n* is the number of elements, *x_i_* is the mole fraction of each element, *T_i_* is the melting temperature of each element, and *T_m_* is the average melting temperature of the *n*-element alloy. *δ* is defined as [22]:

(7) $$\delta =\sqrt{\sum_{i=1}^{n}x_{i}{\left(1-d_{i}/\sum_{j=1}^{n}x_{j}d_{j}\right)}^{2}}\;,$$

where *d_i_* and *d_j_* are the atomic diameters of the components *i* and *j*, respectively.

The electronegativity difference ∆*χ* is defined as [22]:

(8) $$\Delta \chi =\sqrt{\sum_{i=1}^{N}x_{i}{\left(\chi_{i}-\bar{\chi }\right)}^{2}},$$

(9) $$\bar{\chi }=\sum_{i=1}^{N}x_{i}\chi_{i}$$

where $\bar{\chi }$ is the average electronegativity of alloy elements and *χ_i_* is the electronegativity of the *i*-th element of the alloy.

The valence electron concentration (*VEC*) is defined as [22]:

(10) $$VEC=\sum_{i=1}^{N}x_{i}{\left(VEC\right)}_{i},$$

where *(VEC)_i_* is the valence electron concentration of the *i*-th element. The results are shown in Table 1.

After adding Cu and Ag elements with varying contents to the base alloy Fe_63.3_Mn_14_Si_9.1_Cr_9.8_C_3.8_, the entropy value remained between 1R and 1.5R, which is in the range of medium-entropy alloys [23,24]. The alloy-mixing enthalpies were between −19.610 kJ/mol and −14.235 kJ/mol, indicating that the bonding force between the alloy components was strong and that it was easier to form a dense solid solution structure [25,26]. The electronegativity values of the studied medium-entropy alloys varied from 0.180 to 0.185 and were minimal, indicating that the alloys tended to form solid solution structures. The atomic size difference was between 6.391% and 6.841%. The distortion energy in the alloy systems increased with increases in the atomic size differences in the medium-entropy alloy systems.

### 3.2. Microstructure of Fe-Based Medium-Entropy Alloys

**Figure 1:**
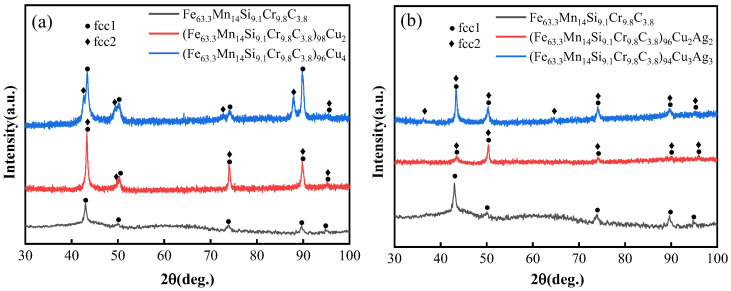
XRD spectra of medium-entropy alloys: (**a**) (Fe_63.3_Mn_14_Si_9.1_Cr_9.8_C_3.8_)_(100−x)_Cu_x_(x = 2,4); (**b**) (Fe_63.3_Mn_14_Si_9.1_Cr_9.8_C_3.8_)_(100−2x)_Cu_x_Ag_x_(x = 2,3).

Figure 1 shows the XRD spectra of (Fe_63.3_Mn_14_Si_9.1_Cr_9.8_C_3.8_)_(100−x)_Cu_x_(x = 2,4) medium-entropy alloys. The Mn and Si elements in the alloys’ main elements could stabilize the austenite phase, and the Mn element could promote austenite growth. The XRD baseline shows the “wavy” shape, which is attributed to an effect arising from lattice distortion in the alloy. Meanwhile, the main peaks present themselves slightly wider due to the rapid cooling of the copper mold, the high entropy effect, and lattice distortion. Among these curves, the peak division of (Fe_63.3_Mn_14_Si_9.1_Cr_9.8_C_3.8_)_98_Cu_2_ was not obvious. Two FCC solid solution phase peaks were observed for (Fe_63.3_Mn_14_Si_9.1_Cr_9.8_C_3.8_)_96_Cu_4_, indicating that the alloy had formed Cu-rich FCC2 phases with significant phase separation from the Fe, Mn, Si, and Cr-rich FCC2 phases. Figure 1b provides the XRD spectra of (Fe_63.3_Mn_14_Si_9.1_Cr_9.8_C_3.8_)_(100−2x)_Cu_x_Ag_x_(x = 2,3) alloys. There is no obvious peak separation between the Cu and Ag-rich phase and the FCC phase rich in Fe, Mn, Si, and Cr for the (Fe_63.3_Mn_14_Si_9.1_Cr_9.8_C_3.8_)_96_Cu_2_Ag_2_ alloy, whereas distinct peaks and significant phase separation are evident for the (Fe_63.3_Mn_14_Si_9.1_Cr_9.8_C_3.8_)_94_Cu_3_Ag_3_ alloy.

**Figure 2:**
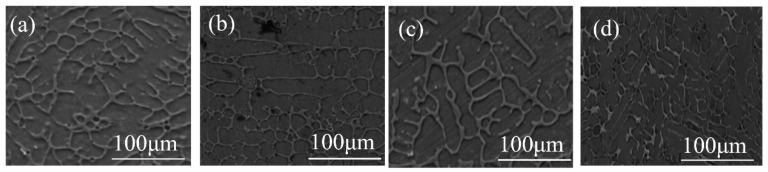
SEM microstructures of medium-entropy alloys (using the SE signals): (**a**) (Fe_63.3_Mn_14_Si_9.1_Cr_9.8_C_3.8_)_98_Cu_2_; (**b**) (Fe_63.3_Mn_14_Si_9.1_Cr_9.8_C_3.8_)_96_Cu_4_; (**c**) (Fe_63.3_Mn_14_Si_9.1_Cr_9.8_C_3.8_)_96_Cu_2_Ag_2_, and (**d**) (Fe_63.3_Mn_14_Si_9.1_Cr_9.8_C_3.8_)_94_Cu_3_Ag_3_.

**Figure 3:**
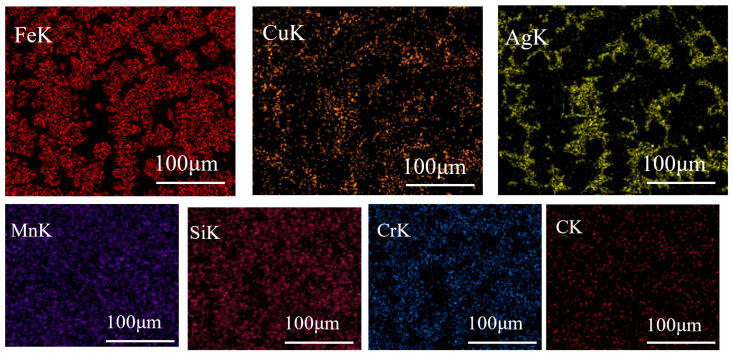
EDS element surface distribution of (Fe_63.3_Mn_14_Si_9.1_Cr_9.8_C_3.8_)_94_Cu_3_Ag_3_ medium-entropy alloy.

Figure 2 shows the microstructures of Fe-based medium-entropy alloys. Under a negative temperature gradient, the grain size increases from the surface to the interior. The faster cooling rate increases undercooling and promotes fine dendrite formation. When Figure 2a,b (in which the Cu content increased from a to b) is compared with the EDS element distribution in Figure 3, it is evident that the enrichment of Cu elements was more prominent; there was a distribution trend among dendrites in which Fe, Mn, Si, Cr, and C elements were alloyed in more uniform distributions in the dendrite region. Figure 2c,d show the microstructure of the (Fe_63.3_Mn_14_Si_9.1_Cr_9.8_C_3.8_)_(100−2x)_Cu_x_Ag_x_(x = 2,3) alloy sample; the elemental distribution in the matrix was relatively uniform, and no inclusions were observed. From the element distribution diagram in Figure 3, it can be observed that Cu and Ag elements were mainly enriched in the intergranular area (the FCC2 phase is lighter in contrast, which is rich in Cu and Ag elements). As the binary mixing enthalpies of Cu, Ag, and the main components Fe, Mn, and Cr in the medium-entropy alloy were all positive, the Cu and Ag elements were repelled to the intergranular area before the melt solidified, so that the Cu and Ag enriched phases (FCC2 phase) were precipitated between crystals, and phase separation occurred. Although the mixing enthalpy between Cu and Ag is positive (as they are elements of cognate elements), they are similar in structure and properties. According to the solid solution theory, Ag will dissolve in Cu and rapidly cool to form an FCC2 phase rich in Cu and Ag. Microscopic phase separation occurs at different scales in the Fe-, Mn-, Cr-, and Si-rich FCC phases.

### 3.3. Corrosion Resistance

**Figure 4:**
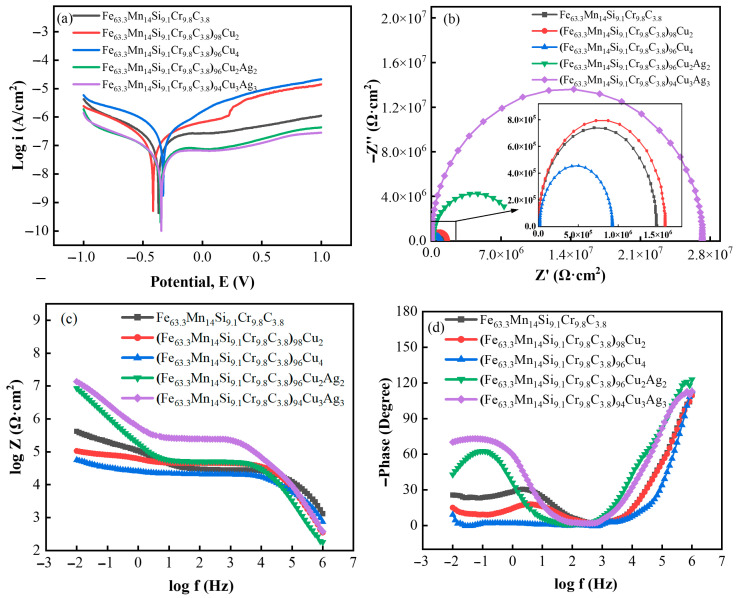
Electrochemical test results of medium-entropy alloys in PBS solution: (**a**) Tafel polarization curves; (**b**) Nyquist plots; (**c**) Bode plots; and (**d**) phase angles.

To better understand the corrosion resistance of medium entropy alloys, the medium entropy alloy Fe_63.3_Mn_14_Si_9.1_Cr_9.8_C_3.8_ and the alloys (Fe_63.3_Mn_14_Si_9.1_Cr_9.8_C_3.8_)_(100−x)_Cu_x_(x = 2,4) and (Fe_63.3_Mn_14_Si_9.1_Cr_9.8_C_3.8_)_(100−2x)_Cu_x_Ag_x_(x = 2,3) were immersed in PBS solutions for corrosion resistance tests. Figure 4a shows the potentiodynamic polarization curves of the alloy in a PBS solution. Combined with Table 2, the corrosion performance of Fe-based medium-entropy alloys in a PBS solution deteriorates with the increase of Cu content. With the simultaneous increase in Cu and Ag content, the corrosion resistance of the Fe-based medium-entropy alloy in PBS solution is improved. The corrosion current density of (Fe_63.3_Mn_14_Si_9.1_Cr_9.8_C_3.8_)_94_Cu_3_Ag_3_ reached 1.357 × 10^−8^ A·cm^−2^ and had excellent corrosion resistance. The combined effect of Cu and Ag makes the corrosion resistance of the Fe-based entropy alloys significantly improved.

To better understand the corrosion resistance of medium-entropy alloys, the medium-entropy alloy Fe_63.3_Mn_14_Si_9.1_Cr_9.8_C_3.8_ and the alloys (Fe_63.3_Mn_14_Si_9.1_Cr_9.8_C_3.8_)_(100−x)_Cu_x_(x = 2,4) and (Fe_63.3_Mn_14_Si_9.1_Cr_9.8_C_3.8_)_(100−2x)_Cu_x_Ag_x_(x = 2,3) were immersed in PBS solutions for corrosion resistance tests. Figure 4a shows the potentiodynamic polarization curves of the alloys in PBS solutions. Considering the results of Table 2, these curves indicate that the corrosion performance of the Fe-based medium-entropy alloys in PBS solution deteriorated as Cu content increased. With a simultaneous increase in Cu and Ag content, the corrosion resistance of Fe-based medium-entropy alloys in PBS solutions improved. The corrosion current density of (Fe_63.3_Mn_14_Si_9.1_Cr_9.8_C_3.8_)_94_Cu_3_Ag_3_ reached 1.357 × 10^−8^ A·cm^−2^ and had excellent corrosion resistance. The combined effect of Cu and Ag significantly improved the corrosion resistance of the Fe-based entropy alloys.

Figure 4b shows the Nyquist spectra of the alloys corroded in PBS solutions. Each alloy’s AC impedance fitting results show a single arc of capacitive reactance. A larger capacitive arc radius corresponded to better corrosion resistance. The Fe-based entropy alloys’ arc radii were arranged as follows: (Fe_63.3_Mn_14_Si_9.1_Cr_9.8_C_3.8_)_94_Cu_3_Ag_3_ > (Fe_63.3_Mn_14_Si_9.1_Cr_9.8_C_3.8_)_96_Cu_2_Ag_2_ > (Fe_63.3_Mn_14_Si_9.1_Cr_9.8_C_3.8_)_98_Cu_2_ > Fe_63.3_Mn_14_Si_9.1_Cr_9.8_C_3.8_> (Fe_63.3_Mn_14_Si_9.1_Cr_9.8_C_3.8_)_96_Cu_4_; the (Fe_63.3_Mn_14_Si_9.1_Cr_9.8_C_3.8_)_94_Cu_3_Ag_3_ alloy exhibited better corrosion resistance.

Figure 4c shows the Bode plots of the alloys in PBS solution; the low-frequency region reflects the resistance of the samples’ passivation films. The alloys’ resistance values, from highest to lowest, were as follows: (Fe_63.3_Mn_14_Si_9.1_Cr_9.8_C_3.8_)_94_Cu_3_Ag_3_, (Fe_63.3_Mn_14_Si_9.1_Cr_9.8_C_3.8_)_96_Cu_2_Ag_2_, (Fe_63.3_Mn_14_Si_9.1_Cr_9.8_C_3.8_)_98_Cu_2_, Fe_63.3_Mn_14_Si_9.1_Cr_9.8_C_3.8_, and (Fe_63.3_Mn_14_Si_9.1_Cr_9.8_C_3.8_)_96_Cu_4_. The phase angles and frequencies are shown in Figure 4d. When an alloy’s phase angle peak was closer to the low-frequency region and larger in value, it indicated that the alloy had good capacitive resistance during the corrosion process and could effectively prevent the intervention of the corrosion medium. The phase angle peak of the (Fe_63.3_Mn_14_Si_9.1_Cr_9.8_C_3.8_)_94_Cu_3_Ag_3_ alloy was larger, indicating that this alloy had excellent corrosion resistance and that adding Ag could improve the corrosion resistance of a medium-entropy alloy. The polarization curve and capacitive reactance arc results were basically the same. In comparison, the corrosion resistance of iron-based medium-entropy alloys in PBS solution was better than it was in 3.5% NaCl solution.

**Figure 5:**
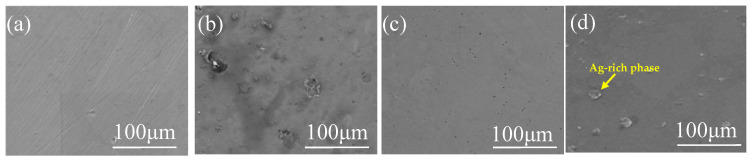
Corrosion morphologies of medium-entropy alloys in PBS solution: (**a**) (Fe_63.3_Mn_14_Si_9.1_Cr_9.8_C_3.8_)_98_Cu_2_; (**b**) (Fe_63.3_Mn_14_Si_9.1_Cr_9.8_C_3.8_)_96_Cu_4_; (**c**) (Fe63.3Mn14Si9.1Cr9.8C3.8)_96_Cu_2_Ag_2_; and (**d**) (Fe63.3Mn14Si9.1Cr9.8C3.8)_94_Cu_3_Ag_3_.

**Figure 6:**
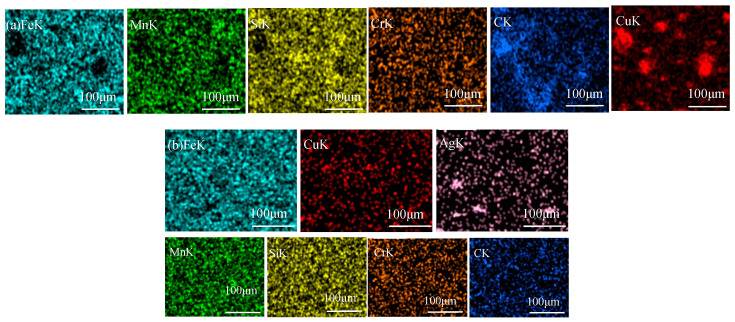
Surface distribution of EDS elements after corrosion of medium-entropy alloys in PBS solution: (**a**) (Fe_63.3_Mn_14_Si_9.1_Cr_9.8_C_3.8_)_96_Cu_4_ and (**b**) (Fe_63.3_Mn_14_Si_9.1_Cr_9.8_C_3.8_)_94_Cu_3_Ag_3_.

Figure 5 shows the surface morphology of medium-entropy alloys after corrosion in PBS solutions. In Figure 5a, the (Fe_63.3_Mn_14_Si_9.1_Cr_9.8_C_3.8_)_98_Cu_2_ alloy’s surface exhibited almost no pits and additional traces of corrosion. However, some corrosion pits and external corrosion appeared on the surface of the (Fe_63.3_Mn_14_Si_9.1_Cr_9.8_C_3.8_)_96_Cu_4_ alloy, as shown in Figure 5b. That is, adding more Cu content decreases the anti-corrosive property. Additionally, as evident in the EDS elemental distribution map in Figure 6a, these corrosion pits were mainly concentrated in the Cu-enriched area. Figure 5c,d show that the (Fe_63.3_Mn_14_Si_9.1_Cr_9.8_C_3.8_)_96_Cu_2_Ag_2_ and (Fe_63.3_Mn_14_Si_9.1_Cr_9.8_C_3.8_)94Cu_3_Ag_3_ alloys had almost no corrosion pits, respectively. Figure 6b provides a surface distribution of EDS elements corresponding to the microstructure in Figure 5. An Ag-rich phase precipitated on the Fe-based MEA matrix and therefore suppressed the tendency for pitting corrosion caused by excessive Cu.

**Figure 7:**
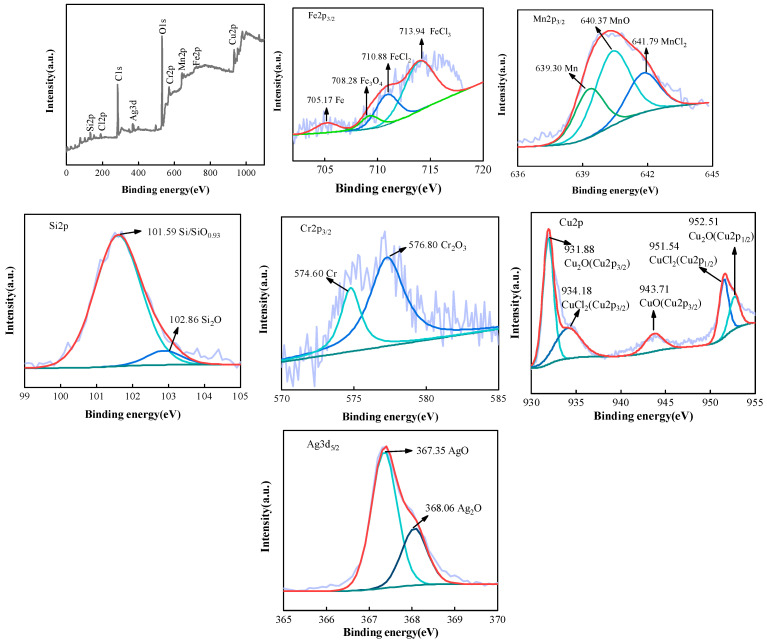
XPS spectrum of the (Fe_63.3_Mn_14_Si_9.1_Cr_9.8_C_3.8_)94Cu_3_Ag_3_ alloy after electrochemical corrosion in PBS solution.

Figure 7 shows the XPS spectrum of the (Fe_63.3_Mn_14_Si_9.1_Cr_9.8_C_3.8_)94Cu_3_Ag_3_ alloy after electrochemical corrosion. The corrosion layer contained Fe, Mn, Cr, O, Ag, C, Cl, Si, and other elements. Among them, Fe, Mn, Cr, Ag, C, and Si were from the alloy matrix, and O and Cl were mainly from the corrosive medium [27,28,29]. The XPS energy spectrum of the Fe2p3/2 alloy had four peaks; the characteristic peaks corresponded to Fe, Fe_3_O_4_, FeCl_2_, and FeCl_3_. The Fe in the passivation film mainly existed in the form of elemental Fe, Fe_3_O_4_, Fe^2+^, and Fe^3+^ chloride, indicating that corrosion had occurred. The characteristic peaks of Mn2p3/2XPS were Mn, MnO, and MnCl_2_, among which MnO’s intensity was the highest, indicating the passivation film’s stability. The characteristic peaks of Si2p corresponded to SiO_0.93_ and Si_2_O. The energy spectrum of Cr2p_3/2_ could be solved as two peaks. The intensity of the characteristic Cr_2_O_3_ peak was higher than that of Cr, which was the main element determining the alloy’s corrosion resistance. In passivated films, Ag mainly existed in the forms of Ag^+^ and Ag^2+^ oxides; the characteristic peak intensity of AgO was higher than that of Ag_2_O, indicating that Ag interacted with external O on the passivation film’s surface and started to transform into high-valence oxides. The Cu2pXPS scan spectrum yielded five characteristic peaks after the spectrum’s resolution, which indicated that the Cu on the specimen’s surface interacted with the Cl^−^ and O_2_ in the solution. The Cu_2_O(Cu2p1/2) peak had the highest intensity; Cu mainly existed as Cu_2_O [30,31].

## 4. Discussion

According to the corrosion resistance results, including the Tafel polarization curves, Bode plots, phase angles and frequencies, and XPS analysis, the cathode reaction of (Fe_63.3_Mn_14_Si_9.1_Cr_9.8_C_3.8_)94Cu_3_Ag_3_ in the corrosion process of PBS solution was:

O_2_ + 2H_2_O + 4e^−^ → 4OH^−^. (11)

Cr was the main element that determined the corrosion resistance in the entropy alloy in the Fe-Mn-Cr-Si system, and it easily acted to form a protective passivation film of Cr_2_O_3_, which could effectively block the movement of alloy ions and effectively stabilize the passivation film to ensure the alloy’s corrosion resistance [32].

2Cr + 3H_2_O → Cr_2_O_3_ + 6H^+^ + 6e^−^. (12)

Fe, Mn, Si, and other elements, due to their low corrosion potential, lost electrons as the anode and were oxidized. As the corrosion potential gradually increased, a passivation film was formed, as shown in the following formulas [33]:

Fe → Fe^2+^ + 2e^−^, (13)

Fe → Fe^3+^ + 3e^−^, (14)

3Fe + 4H_2_O → Fe_3_O_4_ + 8H^+^ + 8e^−^, (15)

Mn → Mn^2+^ + 2e^−^, (16)

Si → Si^2+^ + 2e^−^. (17)

The corrosion product of the Si element in the alloy’s corrosion-product film layer was mainly its oxide, which was dispersed in the Cr_2_O_3_ oxide film, increasing the passivation film’s thickness [34]. The corrosion products of the Fe and Mn elements were dominated by their oxides and chlorides, and a small amount of Fe and Mn ions filled in the inner film layer of the corrosion products. In the early stage of corrosion, the oxides and chlorides formed by Fe and Mn elements were dispersed in the inner film layer of the corrosion product, which increased the passivation film’s thickness and density, hindered the movement of ions, increased the alloy’s electrode potential, and ensured the alloy’s stability [4].

The added Cu elements in the alloy were in direct contact with Cl^−^ in the pre-corrosion stage and underwent an anodic reaction to rapidly generate Cu_2_O and CuCl in the form of dissolution–deposition to cover the alloy’s surface, as shown in the following formulas [35,36]:

Cu^+^ + Cl^−^ → CuCl, (18)

2CuCl + H_2_O → Cu_2_O + 2H^+^ + 2Cl^−^. (19)

At this point, Cu could reduce the corrosion and dissolution of Cr_2_O_3_, increase the passivation film’s thickness, and stabilize the Cr, Mn, and Si oxides in the passivation film, thus protecting it. As the corrosion progressed, the resistance of Cu^+^ and e^−^ to pass through the passivation film increased, the corrosion rate decreased, the cathodic reaction was inhibited, and the anodic reaction continued to occur with H_2_O, O_2_, and Cl^−^ in the solution. The reaction is shown in the following formulas [37]:

2Cu_2_O + O_2_→ 4CuO, (20)

CuCl + Cl^−^ → CuCl_2_ + e^−^, (21)

However, due to the low potential of Cu in the Cu- and Ag-enriched intergranular area, Cu_2_O and CuCl rapidly formed on the alloy’s surface when it was in direct contact with Cl^−^. As the corrosion progressed, the Ag element formed its oxides and dispersed in the passivation film, which increased the passivation film’s density and thickness, reduced the reaction rate, and ensured the alloy’s corrosion resistance [38].

**Figure 8:**
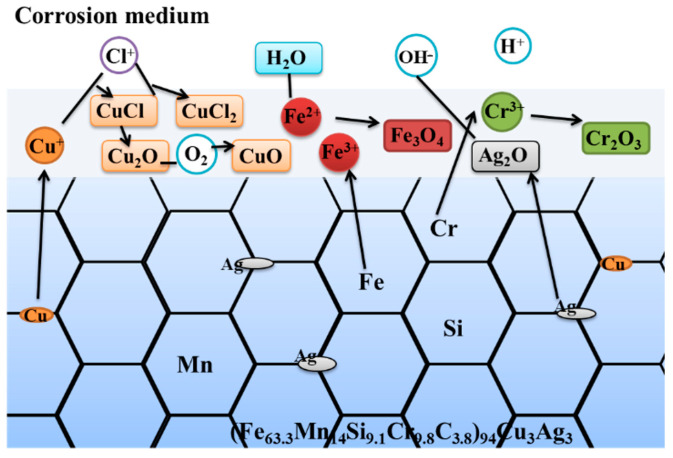
Electrochemical corrosion mechanism of the (Fe_63.3_Mn_14_Si_9.1_Cr_9.8_C_3.8_)_94_Cu_3_Ag_3_ alloy.

In summary, as shown in Figure 8, in the Fe-Mn-Cr-Si medium-entropy alloy, the Cr_2_O_3_ oxide was dense and stable and thus displayed strong passivation ability. The oxides formed by Fe, Si, and Mn could increase the passivation film’s thickness and stability, blocking the movement of alloy ions, to improve corrosion resistance. While the Cu elements were in direct contact with Cl^−^ and dissolved in the early stage of corrosion. Cu_2_O and CuCl were rapidly formed as deposition, which covered the alloy’s surface, increased the passivation film’s thickness, and slowed the reactions of other elemental oxides [39]. However, with the increased Cu content, the alloy presented obvious composition segregation, and a Cu-rich phase appeared at the grain boundary. The fast corrosion rate of Cu provided a channel for Cl^−^, and the grain boundary’s corrosion deepened as a result. However, mild corrosion in the intergranular area protected by Cr_2_O_3_ caused the interdendritic and the dendrite to form the active primary battery, with corrosion mainly concentrated in the interdendritic area. Therefore, the corrosion resistance of the (Fe_63.3_Mn_14_Si_9.1_Cr_9.8_C_3.8_)_96_Cu_4_ alloy decreased significantly.

While Cu and Ag elements were added to the alloy simultaneously, the alloy’s mixing entropy ∆S_mix_ and lattice distortion increased (due to the Cu and Ag alloying), which improved the alloy’s corrosion resistance. In the early stage of corrosion, the Ag element promoted a more uniform and refined microstructure, and the Cu^2+^ that precipitated in the alloy dissolved in the anode, which could stabilize the passivation film. With the corrosion time increasing, the dense oxide formed by Ag provided the alloy’s passivation film with the ability to become continuous and stable. Compared with Co_1−x_CrFeNi_1+x_Cu_y_ HEA [13] and Cu_x_Cr_2_Fe_2_Ni_3_Mn_2_Nb_0.4_Mo_0.2_ (x = 0,0.1,0.2) [14], the synergistic effect of Ag and Cu elements ensured the (Fe_63.3_Mn_14_Si_9.1_Cr_9.8_C_3.8_)94Cu_3_Ag_3_ alloy had excellent corrosion resistance.

## 5. Conclusions

In this study, the effects of adding different Ag and Cu contents to the alloy on the alloy’s corrosion resistance were studied, and the main conclusions are as follows:

(1) After adding different proportions of Cu and Ag elements, the entropy value of the alloy was still in the range of entropy alloys (from 1R to 1.5R); the mixing enthalpy of the alloy was all negative. Regarding the alloy’s morphology, it was observed that Cu and Ag elements were enriched in the intergranular area, as the binary mixing enthalpies of Cu and Ag and the main elements Fe, Mn, Si, and Cr of the medium-entropy alloy were all positive;
(2) With an increase in Cu and Ag content, the alloy’s corrosion potential increased, the corrosion current density decreased, the radius of the capacitive arc gradually increased, and the alloy’s corrosion resistance was enhanced. The corrosion current density of (Fe_63.3_Mn_14_Si_9.1_Cr_9.8_C_3.8_)94Cu_3_Ag_3_ in PBS solution reached 1.357 × 10^−8^ A·cm^−2^;
(3) The synergistic effect of Cu and Ag elements made the alloy exhibit excellent corrosion resistance. Si, Cr, Ag, and other elements in the corrosion product film were combined with electron holes to fill in the corrosion in the product lattice; the passivation film’s conductivity was reduced, the corrosion rate slowed, and the alloy’s corrosion resistance was guaranteed.

## Figures and Tables

**Table 1:** Thermodynamic parameters of medium-entropy alloys.

Alloys	∆*S_mix_* (J·mol^−1^·K^−1^)	∆*H_mix_* (kJ/mol)	*Ω*	*δ* (%)	*∆χ*	*VEC*
Fe_63.3_Mn_14_Si_9.1_Cr_9.8_C_3.8_	1.141R	−19.610	0.903	6.720	0.184	7.141
(Fe_63.3_Mn_14_Si_9.1_Cr_9.8_C_3.8_)_98_Cu_2_	1.215R	−18.471	1.017	6.721	0.185	7.218
(Fe_63.3_Mn_14_Si_9.1_Cr_9.8_C_3.8_)_96_Cu_4_	1.255R	−16.705	1.152	6.391	0.180	7.302
(Fe_63.3_Mn_14_Si_9.1_Cr_9.8_C_3.8_)_96_Cu_2_Ag_2_	1.284R	−15.824	1.242	6.692	0.180	7.302
(Fe_63.3_Mn_14_Si_9.1_Cr_9.8_C_3.8_)_94_Cu_3_Ag_3_	1.336R	−14.235	1.431	6.841	0.181	7.379

**Table 2:** Performance parameters of potentiodynamic polarization curves of medium-entropy alloys in PBS.

Alloy	*E_corr_* (V)	*I_corr_* (A·cm^−2^)	*R* (Ω·cm^2^)
Fe_63.3_Mn_14_Si_9.1_Cr_9.8_C_3.8_	−0.370	8.089 × 10^−8^	5.7238 × 10^5^
(Fe_63.3_Mn_14_Si_9.1_Cr_9.8_C_3.8_)_98_Cu_2_	−0.415	8.332 × 10^−8^	5.2500 × 10^5^
(Fe_63.3_Mn_14_Si_9.1_Cr_9.8_C_3.8_)_96_Cu_4_	−0.331	1.960 × 10^−7^	2.3082 × 10^5^
(Fe_63.3_Mn_14_Si_9.1_Cr_9.8_C_3.8_)_96_Cu_2_Ag_2_	−0.353	1.659 × 10^−8^	2.8842 × 10^6^
(Fe_63.3_Mn_14_Si_9.1_Cr_9.8_C_3.8_)_94_Cu_3_Ag_3_	−0.346	1.357 × 10^−8^	3.4824 × 10^6^

## References

[1] Lo K.H., Shek C.H., Lai J.K.L. (2009). Recent developments in stainless steels. Mater. Sci. Eng. R.

[2] Shi Y.Z., Yang B., Liaw P. (2017). Corrosion-Resistant High-Entropy Alloys: A Review. Metals.

[3] Yao Q., Sebastian T., Mark A.G., Hamish L.F., Nick B. (2017). Corrosion of high entropy alloys. NPJ Mater. Degrad..

[4] Salishchev G.A., Tikhonovsky M.A., Shaysultanov D.G., Stepanov N.D., Kuznetsov A.V., Kolodiy I.V., Tortika A.S., Senkov O.N. (2014). Effect of Mn and V on structure and mechanical properties of high entropy alloys based on CoCrFeNi system. J. Alloys Compd..

[5] Han J.S., Su B., Meng J.H., Zhang A.J., Wu Y.Z. (2020). Microstructure and composition evolution of a fused slurry silicide coating on MoNbTaTiW refractory high-entropy alloy in high temperature oxidation environment. Materials.

[6] Laplanche G., Kostka A., Reinhart C., Hunfeld J., Eggeler G., Georgeet E.P. (2017). Reasons for the superior mechanical properties of medium entropy CrCoNi compared to high entropy CrMnFeCoNi. Acta Mater..

[7] Zhao Y.C., Ma H.W., Zhang L.H., Zhang D., Kou S.Z., Wang BLi W.S., Liaw P.K. (2022). Mn content optimum on microstructures and mechanical behavior of Fe-based medium entropy alloys. Mater. Des..

[8] Shi P., Li R., Li Y., Wen Y., Zhong Y., Ren W., Shen Z., Zheng T., Peng J., Liang X. (2021). Hierarchical crack buffering triples ductility in eutectic herringbone high-entropy alloys. Science.

[9] Wang Y.Z., Jiao Z.M., Bian G.B., Yang H.J., He H.W., Wang Z.H., Liaw P.K., Qiao J.W. (2022). Dynamic tension and constitutive model in in Fe_40_Mn_20_Cr_20_Ni_20_ highentropy alloys with a heterogeneous structure. Mater. Sci. Eng. A.

[10] Lu C.W., Lu Y.S., Lai Z.H., Yen H.W., Lee Y.L. (2020). Comparative corrosion behavior of Fe_50_Mn_30_Co_10_Cr_10_ dual-phase high-entropy alloy and CoCrFeMnNi high-entropy alloy in 3.5wt% NaCl solution. J. Alloys Comd..

[11] Guo W., Su J., Lu W., Liebscher C.H., Kirchlechner C., Ikeda Y., Körmann F., Liu X., Xue Y., Dehm G. (2020). Dislocation-induced breakthrough of strength and ductility trade-off in a non-equiatomic high-entropy alloy. Acta Mater..

[12] Gao M.C., Qiao J.W. (2018). High-Entropy Alloys (HEAs). Metals.

[13] Liu F., Song Q., Chen R., Wang C., Sun J. (2023). Effect of Co, Ni, Cu content on phase composition, microstructure and corrosion resistance of Co_1−x_CrFeNi_1+x_Cuy series high-entropy alloys. Vacuum.

[14] Li J.C., Liu Z.X., Chen Y.D., CAO Y.J. (2018). Microstructure and Corrosion Behavior of CuxCr_2_Fe_2_Ni_3_Mn_2_Nb_0_._4_Mo_0_._2_ High Entropy Alloy. Spec. Cast. Nonferrous Alloys.

[15] Hsu Y.J., Chiang W.C., Wu J.K. (2005). Corrosion behavior of FeCoNiCrCu_x_ high-entropy alloys in 3.5% sodium chloride solution. Mater. Chem. Phys..

[16] Gaskell D.R. (2008). Introduction to the Thermodynamics of Materials.

[17] Swalin R.A. (1972). Thermodynamics of Solids.

[18] Gao M.C., Yeh J.W., Liaw P.K., Zhang Y. (2016). High-Entropy Alloys: Fundamentals and Applications.

[19] Yang X., Zhang Y. (2012). Prediction of high-entropy stabilized solid-solution in multicomponent alloys. Mater. Chem. Phys..

[20] Zhang Y., Yang X., Liaw P.K. (2012). Alloy design and properties optimization of high-entropy alloys. JOM.

[21] Yang X., Chen S.Y., Cotton J.D., Zhang Y. (2014). Phase stability of low-density, multiprincipal component alloys containing aluminum, magnesium, and lithium. JOM.

[22] Zhang Y., Zhou Y.J., Lin J.P., Chen G.L., Liaw P.K. (2008). Solid-solution phase formation rules for multi-component alloys. Adv. Eng. Mater..

[23] Chen R., Qin G., Zheng H., Wang L., Su Y., Chiu Y., Ding H., Guo J., Fu H. (2018). Composition design of high entropy alloys using the valence electron concentration to balance strength and ductility. Acta Mater..

[24] Zhou Y., Zhou D., Jin X., Zhang L., Du X., Li B. (2018). Design of non-equiatomic medium-entropy alloys. Sci. Rep..

[25] Ding Q., Zhang Y., Chen X., Fu X., Chen D., Chen S., Gu L., Wei F., Bei H., Gao Y. (2019). Tuning element distribution, structure and properties by composition in high-entropy alloys. Nature.

[26] Zhevnenko S.N., Chernyshikhin S.V. (2017). Surface Phase Transitions in Cu-Based Solid Solutions. Appl. Surf. Sci..

[27] Huang J., Li Z., Liaw B.Y., Zhang J.B. (2016). Graphical analysis of electrochemical impedance spectroscopy data in Bode and Nyquist representations. J. Power Sources.

[28] Liang C.H., Liu L.B., Jia Z., Dai C.S., Xiong Y.P. (2015). Synergy of Nyquist and Bode electrochemical impedance spectroscopy studies to particle size effect on the electrochemical properties of LiNi_0.5_Co_0.2_Mn_0.3_O_2_. Electrochim. Acta.

[29] Monaco L., Sodhi R.N., Palumbo G., Erb U. (2020). XPS study on the passivity of coarse-grained polycrystalline and electrodeposited nanocrystalline nickel-iron (NiFe) alloys. Corros. Sci..

[30] Fajardo S., Llorente I., Jiménez J.A., Bastidas J.M., Bastidas D.M. (2019). Effect of Mn additions on the corrosion behaviour of TWIP Fe-Mn-Al-Si austenitic steel in chloride solution. Corros. Sci..

[31] Kim Y., Buchheit R.G., Kotula P.G. (2010). Effect of alloyed Cu on localized corrosion susceptibility of Al–Cu solid solution alloys—Surface characterization by XPS and STEM. Electrochim. Acta.

[32] Stephan-Scherb C., Menneken M., Weber K., Jácome L.A., Nolze G. (2020). Elucidation of orientation relations between Fe-Cr alloys and corrosion products after high temperature SO_2_ corrosion. Corros. Sci..

[33] Liu L., Chen J.H., Wang S.B., Liu C.H., Yang S.S., Wu C.L. (2014). The effect of Si on precipitation in Al–Cu–Mg alloy with a high Cu/Mg ratio. Mater. Sci. Eng. A.

[34] Liu G., Song H., Feng L., Du X., Liu J. (2021). Corrosion of Fe–B–Si alloys in liquid zinc. Mater. Chem. Phys..

[35] Yang L., Shi Y., Shen L., Zhang E., Qin G., Lu X., Zhou X. (2021). Effect of Ag on cathodic activation and corrosion behaviour of Mg-Mn-Ag alloys. Corros. Sci..

[36] Stansbury E.E., Buchanan R.A. (2000). Fundamentals of Electrochemical Corrosion.

[37] Cao Z.Q., Yin X.T., Jia Z.Q., Tian Q.Y., Jie L.U., Zhang K., Yan W.A. (2019). Corrosion behavior of bulk two-phase Ag−25Cu alloys with different microstructures in NaCl aqueous solution. Trans. Non. Metals Soc. China.

[38] Yu L., Zhao Z., Tang C., Li W., You C., Chen M. (2020). The mechanical and corrosion resistance of Mg-Zn-Ca-Ag alloys: The influence of Ag content. J. Mater. Res. Technol..

[39] Deschamps A., Militzer M., Poole W.J. (2001). Precipitation Kinetics and Strengthening of a Fe-0.8%Cu Alloy. ISIJ Int..

